# How the Chinese Government Has Done with Public Health from the Perspective of the Evaluation and Comparison about Public-Health Expenditure

**DOI:** 10.3390/ijerph17249272

**Published:** 2020-12-11

**Authors:** Hui Jin, Xinyi Qian

**Affiliations:** 1 Department of Economics, School of Economics and Management, Zhejiang Sci-Tech University, Hangzhou 310018, China; 2 Department of Foreign Languages, Qianjiang College, Hangzhou Normal University, Hangzhou 310036, China; 1111704021@zjut.edu.cn

**Keywords:** public-health expenditure, Chinese case, health affairs, health policy

## Abstract

The COVID-19 epidemic has crashed on the social and economic stability of China and even the world, and raised the question: how has the Chinese government done with public health in recent years? The purpose of this paper is to clarify the definition and items of Chinese public-health expenditure, then to objectively evaluate the Chinese government’s performance, so as to help the government to perform better in public health. To achieve this goal, we measure the Chinese public-health expenditure at national and provincial levels based on our definition, and then compare it with the expenditures of other countries. The results show that: (1) the level of public-health expenditure in China is relatively low and far lower than that in developed countries; (2) Chinese governments have not paid enough attention to the prevention and control of major public-health emergencies, which may be an important reason for the outbreak of COVID-19; (3) Chinese public-health expenditure shows a fluctuating growth trend, but the growth rate is so slow that it is lower than that of GDP and fiscal expenditure; (4) although the Chinese government inclines the public-health expenditure to the poor provinces in central and western regions, the imbalance and inequity of public-health resource allocation are still expanding among provinces; (5) there is a lot of waste of resources in the public-health system, which seriously reduces the efficiency of public-health expenditure in China. Therefore, the Chinese government should improve the quantity and quality of public-health expenditure in the above aspects.

## 1. Introduction

The COVID-19 epidemic has stirred a fervent debate worldwide and become the media buzz topic over the recent year. The pandemic, however, has aroused discontent among Chinese masses at the outset, and it has even been exaggerated as “the Chinese version of Chernobyl” [1], specifically in terms of the deficient supervision and information disclosure of the Chinese government. In particular, the adversity of Dr. Li Wenliang has triggered a long torrent of anger in China. Dr. Li Wenliang had posted the information of novel coronavirus pneumonia on the Internet when the epidemic had not yet spread, but he was punished by the police for the reason of “spreading rumors”. Unfortunately, he contracted serious novel coronavirus pneumonia when he received his patients, and died after all-out efforts to save him on 7 February 2020. He is known as the “whistleblower” of the epidemic because of the protection warning. Finally, Dr. Li Wenliang was rated as a martyr by the Chinese government [2]. In addition, the epidemic has inflicted the retail, catering, real estate, tourism, media, and entertainment industries to a great extent, leading to economic decline for several consecutive months in China. Admittedly, it has caused more than 50 million infections and 1 million deaths by November 2020 worldwide, and the number of mortalities is continuously climbing [3]. Therefore, it is safe to say that the pandemic is the culprit of a depression involving politics, economy, and society all over the world [4]. Here comes the question: how has the Chinese government done in the field of public health?

Public-health expenditure is regarded as one of the most important indicators by which to examine the government’s performance in public health [5,6]. The public health market will fail because of the externality, which will lead to insufficient supply of public-health services and goods [7]. So, the government must intervene in public-health market through its public-health system and policy: (1) to try to ensure that the supply of public health goods is adequate and optimal [8]; (2) to justify the efficiency and equity of public health and medical insurance services [9]; (3) and to help the poor get access to basic health care services [10,11]. Clearly, the public-health system serves as a dominant pillar in social development [12,13]. And the operation of the public-health system and the implementation of public health policies are mainly reflected by the government’s public-health expenditure [14,15,16]. As Dominique [17] has pointed, the government plays an important role in improving social welfare, while fiscal expenditure is the key to public goods and services. Cutler and Miller [18] and Verma et al. [19] also point that public-health expenditure reflects the government’s public health policy and it is a contributing factor in maintaining the national health. Therefore, many researchers have carried out a serious of studies on public-health expenditure in various countries, such as the United States [20], Canada [21], Spain [22], China [6], India [23], and the United Kingdom [24]. In conclusion, it is of great significance to study the public-health expenditure for the development of public-health system and policy.

Certainly many literatures have shed a spotlight on the definition and measurement of Chinese public-health expenditure. Generally, Chinese public-health expenditure is divided into narrow-caliber and wide-caliber public-health expenditure [25]. The narrow-caliber public-health expenditure (NPHE) refers to expenditure on pure public-health goods such as those relating to disease surveillance and control, hygiene supervision, public health research, health policy development, and so on. The wide-caliber public-health expenditure (WPHE) adds the expenditure of quasi-public goods, such as medical services, based on the NPHE. Some researchers tend to interpret Chinese public-health expenditure in terms of NPHE, like E and Liu [26]. But some suggest that the WPHE is more scientific and reasonable, like Wang and Chen [25], Yu et al. [6], and so on.

However, the inconsistent definitions of public-health expenditure will not only cause misuse and mixing of concepts, but also statistical deviation, leading to wrong results [27,28,29]. Even if some researchers have accepted the same caliber definition, such as wide-caliber, the items of public-health expenditure defined by them are still different. For example, Wang and Chen [25] and Yu et al. [6] all accept the wide-caliber definition, but the items of their WPHEs are different. Specifically, the WPHE defined by Wang and Chen [25] includes family planning expenditure, but that defined by Yu et al. [6] does not include this item. It implies “one thousand researchers, one thousand kinds of public-health expenditure”, as well as the different measurements and evaluations of Chinese public-health expenditure. Most studies believe that the level of public-health expenditure in China is too low to meet the needs of nationals, such as those by Ho and Gostin [30]. But Wang [27] points out that the insufficiency of public-health expenditure has been greatly alleviated in China, and the public-health expenditure scale in some provinces even exceeds that of developed countries. They have obtained quite different results in the measurement and evaluation of Chinese public-health expenditure.

Therefore, the purpose of this study can be illustrated as follows. Firstly, this paper tries to justify the definition and items of Chinese public-health expenditure. There is still no clear definition on the concept and items of Chinese public-health expenditure in existing literatures, which leads to conceptual misuse and measurement errors. Secondly, we have made an evaluation of Chinese government performance in public health objectively, through accurately measuring public-health expenditure in national and provincial levels, and the international comparative analysis. The existing literatures mainly focus on the determinants and impact effects of public-health expenditure, but there are few studies like this paper [22,28]. Thirdly, this paper puts forward policy suggestions for the Chinese government to perform better in public health. Fourthly, this paper provides a Chinese lesson and case for the development of public-health systems and policies in various countries.

The rest of the paper is organized as follows. Section 2 clarifies the definition and items of Chinese public-health expenditure. Section 3 measures Chinese public-health expenditure in national and provincial levels, and compares it with that of other countries. Section 4 is evaluation and analysis. Section 5 is conclusions.

## 2. Chinese Public-Health Expenditure

**Figure 1:**
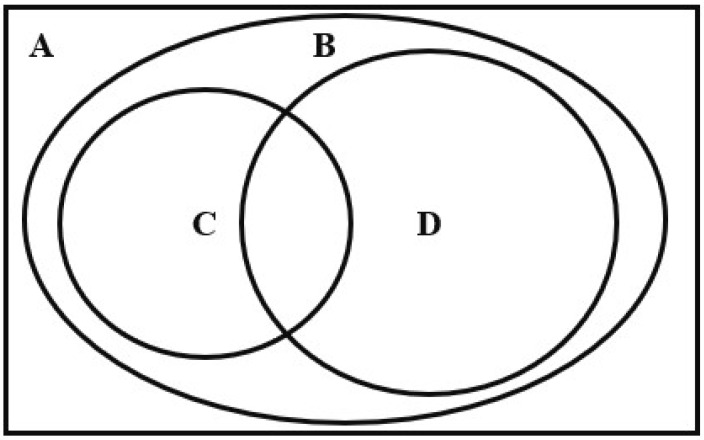
The relationship and difference in different definitions of public-health expenditure. Note: **A** indicates the national health spending (the first type of public-health expenditure), **B** indicates total government health spending (the second type), **C** indicates the narrow-caliber public-health expenditure (the third type), **D** indicates the medical service expenditure, **C** + **D** indicates the wide-caliber public-health expenditure (the fourth type).

The definition of public-health expenditure in existing literatures could be classified into four categories (the relationships and differences between them are shown in Figure 1). The first type of public-health expenditure, also known as the national health spending, represents the total spending of a country (or region) on medical care and public health in a certain period [31,32,33,34]. The second type refers to the total medical care and public health spending by governments [35,36]. (The first type includes the total expenditure of private sectors, social groups, and governments in health care (as the Rectangle A shown in Figure 1), but the second type refers to the government’s expenditure in this field only. So, the second type is also called total government health spending, represented as Oval B shown in Figure 1. The total government health spending of China includes not only public health and medical services items, but also family planning, and other items. Among the items of total government health spending, the spending for public health supervision, improvement of residents’ health status, optimization of the public health environment, and prevention and control of infectious diseases is narrow-caliber public-health expenditure, as Oval C shows; while the spending on the medical care services, institutions, and insurance is called medical service expenditure, as Oval D shows. The narrow-caliber public-health expenditure (NPHE), indicated by Oval C, is the third type. And the fourth type is indicated by C + D, namely the wide-caliber public-health expenditure (WPHE).

In order to avoid statistical errors caused by the inconsistence of definition and measurement caliber, we must clarify the definition and content of public-health expenditure [28,37]. At first, public-health expenditure could be literally understood as the spending on “public health”, while the phrase “public health” implies the public goods or services provided by the government [25]. But the national health spending, namely the first type of public-health expenditure indicated by Rectangle A, includes the spending of government, private, and social groups, which is obviously beyond the conceptual category of “public goods”. Hence, it is not accurate and scientific to define public-health expenditure as national health spending. Secondly, as Winslow’s [38] well-known definition presented, public health is a science and art of preventing diseases, prolonging life, and promoting physical and mental health by formulating policies and systems for the sanitation of the environment, the control of community infections, the education of personal hygiene principles, and the organization of medical and nursing services. The total government health spending of China, namely the second type of public-health expenditure indicated by Oval B, contains some projects that do not conform to the above definition, like family planning expenditure. So, it is not suitable to define public-health expenditure as total government health spending in China. Thirdly, the NPHE also does not conform to Winslow’s definition because it strips out the medical service expenditure. In fact, governmental medical institutions also undertake the important responsibility of public-health services, such as the prevention and control of infectious diseases. As is known to all, Chinese public medical institutions have played a huge role in COVID-19 epidemic prevention and control. If the NPHE is adopted, it will greatly underestimate the functional scope and capital investment of the government in public health.

**Figure 2:**
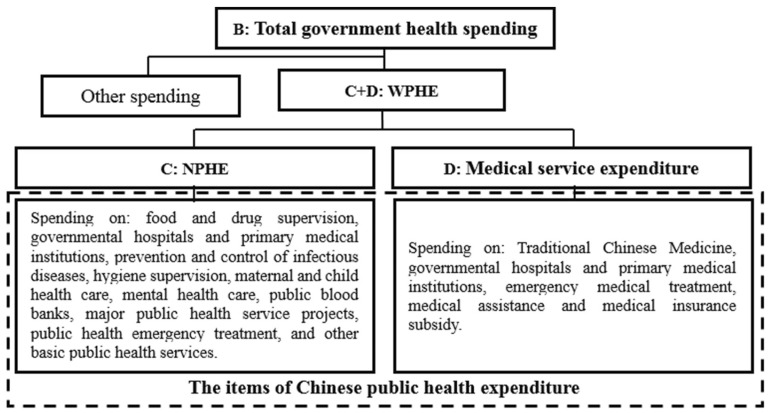
The content of different definitions of public-health expenditure. Note: The implications of **B**, **C**, and **D** in this figure are consistent with those in Figure 1. WPHE is short for wide-caliber public-health expenditure, NPHE is short for narrow-caliber public-health expenditure.

Therefore, it is reasonable and scientific to define the Chinese public-health expenditure as WPHE (if there is no special explanation, the public-health expenditure discussed below refers to the WPHE). According to the definition and Chinese public-health accounts, the items of Chinese public-health expenditure could be settled (see Figure 2). In addition, some studies, such as Wang and Chen [25], consider the family planning project as an item of Chinese public-health expenditure, which has a lack of theoretical and practical basis. Firstly, family planning projects can’t be used as a public health good or service to intervene in health-market failure. Secondly, family planning is unable to help achieve basic public-health goals such as disease prevention, physical and mental health, life prolongation, and so on. Thirdly, most countries do not have family-planning programs, or have not incorporated family planning into the public-health system. Finally, this paper has clarified the relationship and difference of four types of public-health expenditure, and the definition of Chinese public-health expenditure (see Table 1 and Table 2, Figure 1 and Figure 2).

## 3. The Measurement and Comparison of Chinese Public-Health Expenditure

### 3.1. Method and Data

**Figure 3:**
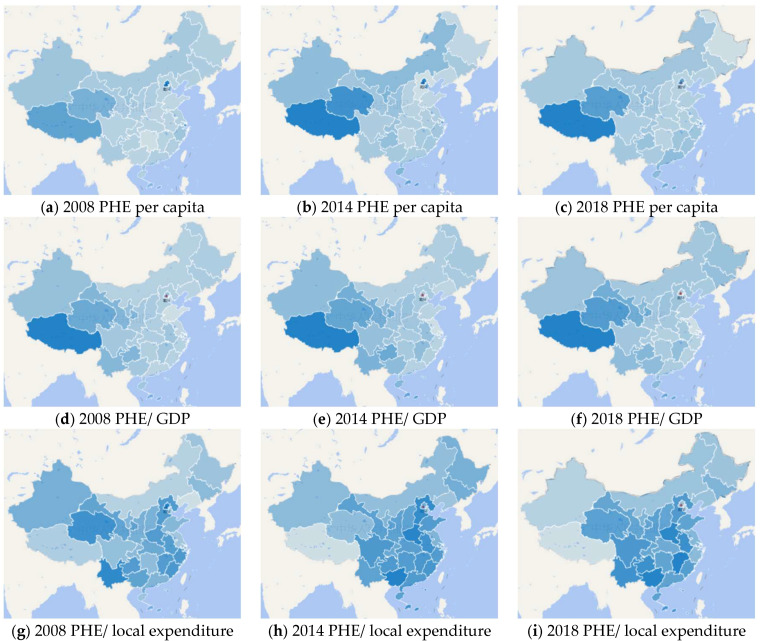
Geographical distribution of Chinese public-health expenditure. Note: PHE is short for public-health expenditure. Note: These figures above are made by the authors, and there is no copyright issue.

This paper paves the way for the objective evaluation of the Chinese government’s performance in public health in next section, through the accurate measurement of Chinese public-health expenditure and the international comparison. Firstly, this paper measures the Chinese public-health expenditure at national and provincial levels. According to the definition above, this paper measures the Chinese public-health expenditure, per capita public-health expenditure, the proportion of public-health expenditure in GDP, and public-health expenditure in total fiscal expenditure from 2014 to 2018. At the same time, this paper lists the specific items and data of Chinese public-health expenditure. After that, this paper measures the per capita public-health expenditure, the proportion of public-health expenditure in provincial GDP, and the proportion of public-health expenditure in local government expenditure in 31 provinces. For simplicity, “province” here is used to indicates municipalities directly under the central government (i.e., Shanghai Municipality, Beijing Municipality) and autonomous regions (i.e., Tibet Autonomous Region) in China. Figure 3 is a geographical distribution map, to aid understanding of the distribution of public-health expenditure in various regions of China.

Secondly, this paper measures the per capita public-health expenditure, the proportion of public-health expenditure in GDP, and the proportion of public-health expenditure in total fiscal expenditure in different countries for international comparison. Six countries are chosen for comparative analysis, including three developed countries and three developing countries, namely the United States, the United Kingdom, Japan, Russia, India, and Thailand (see Table 3). The United States and the United Kingdom are two of the countries with the highest degrees of medical-service marketization and the most effective public-health systems. Japan, a developed country in Asia, is considered by the World Health Organization to have one of the most effective medical systems in the world. As a populous and developing country in the world, India has similar basic national conditions to China, but it began to popularize basic health care for all nationals in the 1950s. Russia is a transitional country, which has similarities with China, but Russia has achieved free medical care for all nationals. Thailand is also a developing country. Although the economic development of Thailand is not as good as that of China, its medical system is well-known in the world, which is worthy of reference. Therefore, the above six countries have been selected as the samples for comparative analysis.

The data for each country come from different databases and statistical documents. The data of Chinese public-health expenditure and fiscal expenditure is from the Finance Yearbook of China (2009, 2015–2019), and the Finance Yearbook of each province in 2009, 2015, and 2019. The data of GDP and population in China is from the China National Bureau of Statistics. The data of the United States’ public-health expenditure and fiscal expenditure is from the president’s Budget of the US Government, the population data is from the Census Bureau, and the GDP data is from the US Department of Commerce. The data of the United Kingdom is from the database of the UK Treasury and public expenditure statistical analyses. The data of India is from the database of India Ministry of Finance. The data of Japan is from the Statistics Bureau of Japan. The data of Thailand comes from the Bank of Thailand. The data of Russian public-health expenditure and government expenditure is from the database of Russian Ministry of Finance, while the data of population and GDP is from the Russian Statistics Bureau.

### 3.2. The Measurement of Chinese Public-Health Expenditure

#### 3.2.1. National Level

Table 1 reports Chinese public-health expenditure and its specific items. Chinese public health-expenditure was 864.75 billion yuan, 1030.87 billion yuan, 1153.24 billion yuan, 1109.20 billion yuan, and 1202.05 billion yuan respectively from 2014 to 2018. Both total and per capita Chinese public-health expenditure show a trend of fluctuating growth. The proportion of public-health expenditure in GDP and fiscal expenditure have experienced the process of first growth and then decline. Specifically, Chinese public-health expenditure includes spending on: governmental hospitals and primary medical institutions, prevention and control of infectious diseases, hygiene supervision, maternal and child health care, mental health care, emergency medical treatment, public blood banks, major public-health service projects, public health emergency treatment, traditional Chinese medicine, food and drug supervision, medical insurance subsidy (i.e., governmental medical insurance, but not private insurance), medical assistance, and other basic public-health services. Among these items, the medical insurance subsidy costs the most, accounting for more than 45% of total public-health expenditure on average; mental health and public-health emergency treatment are the two items with the least expenditure, both accounting for less than 1% of public-health expenditure on average.

#### 3.2.2. Provincial Level

Table 2 reports the measurement of public-health expenditure in 31 provinces, which is including the central government’s fiscal transfer payments to provincial governments. It should be noted that the Chinese government has carried out a comprehensive reform of the public-health system in 2009, to alleviate the imbalance and inequality in the supply of public-health services among regions. Therefore, this paper has included the provincial public-health expenditure data of 2008 for comparison.

The per capita public-health expenditure of 31 provinces is shown in columns 2–4 of Table 2. In respect of regional difference, the per capita public-health expenditure of eastern coastal and western provinces is relatively high, such as Tibet, Beijing, Qinghai, and Shanghai; Heilongjiang, Liaoning, and Shandong are the three provinces with the lowest per capita public-health expenditure, all of which are distributed in northeast China. The per capita public-health expenditures of 31 provinces have all increased from 2008 to 2018. Among these provinces, Hainan, Jiangxi, and Guizhou have the largest increase rate, which have increased more than four times in this period.

The proportion of public-health expenditure in provincial GDP of each province is shown in columns 5–7 of Table 2. The provinces with the highest proportion of public-health expenditure in provincial GDP are mainly distributed in western regions, such as Tibet and Qinghai; the lowest provinces are mainly distributed in the eastern coastal regions, such as Shandong, Jiangsu, and Zhejiang. During this period, the proportion of public-health expenditure in provincial GDP increased in most provinces except Beijing.

The share of public-health expenditure in local fiscal expenditure is shown in 8–10 columns of Table 2. The provinces with high proportion of public-health expenditure in local expenditure are mainly concentrated in central region, such as Jiangxi and Henan. From 2008–2018, the proportion of public-health expenditure in local expenditure have experienced a process of increasing first and then decreasing in 19 provinces, such as Hebei, Jiangsu, Fujian, and so on, which are mainly distributed in the southeast coastal and central regions; 8 provinces are on the rise, such as Inner Mongolia, Ningxia, and other relatively poor provinces; and 4 provinces have showed a downward trend, namely Beijing, Shanghai, Xinjiang, and Tibet.

In order to intuitively show the changes of public-health expenditure of 31 provinces in geography and time dimensions in 2008, 2014, and 2018, this paper has made the geographic distribution map (as shown in Figure 3). It should be noted here that the darker the blue, the higher the level of public-health expenditure. According to (a), (b) and (c) in Figure 3, the provinces with relatively high per capita public-health expenditure are mainly in the western and southeast coastal regions, while this item of the provinces in central region is relatively low. As (d), (e) and (f) in Figure 3 present, the proportion of public-health expenditure in GDP is decreasing from the west to the east. In addition, the proportion of public-health expenditure in local expenditure is relatively high in central regions and relatively low in surrounding provinces.

However, it is worth noting that the results in tables and graphs are not consistent with the actual situation of public-health resource allocation among provinces in China. As these results have shown, Chinese public-health expenditure is inclined to the provinces in central and western regions, especially Tibet and Qinghai. In fact, the public-health resources of rich provinces in eastern coastal region, such as Beijing, Shanghai, and Zhejiang, are much more abundant than those of the central and western provinces. This issue will be explained in detail in Section 4.

### 3.3. The International Comparison of Public-Health Expenditure

Table 3 reports the public-health expenditure of seven countries in 2014 and 2018. Overall, the average level of public-health expenditure in developed countries is much higher than that in developing countries. The per capita public-health expenditure of the three developed countries is all more than 2800 US dollars, while that of the developing countries is less than 500 US dollars. Among these countries, the United States has the highest level of public-health expenditure, and its per capita public-health expenditure has exceeded US $4000 in 2014 and 2018. The United States is also the country with the highest proportion of public-health expenditure in GDP. On the contrary, India has the lowest level of public-health expenditure, accounting for 19 US dollars per capita and 1.18% of fiscal expenditure in 2014. The Japanese government attaches the highest importance to public-health affairs. Its public-health expenditure accounted for more than 36% of fiscal expenditure in 2014 and 2018, which is about seven times that of China and India. In addition, most sample countries have significantly increased their public-health expenditure levels from 2014–2018, except for China. Chinese per capita public-health expenditure increased from 103 to 130 US dollars, but the proportion of public-health expenditure in fiscal expenditure and GDP decreased slightly during this period.

## 4. The Evaluation and Analysis of Chinese Public-Health Expenditure

Considering the realities in China, this paper will objectively evaluate and analyze the performance of the Chinese government in public health, based on the measurement results and international comparison unfolded above. The performance of Chinese government in public health could be roughly summed up in the following six points.

(1) The scale of public-health expenditure in China is quite low, and pales in comparison with that in developed countries. As the 2014 data in Table 3 shows, the per capita public-health expenditure in China is 103 US dollars, about 1/40 of that in the United States, 1/28 in Japan, and 1/34 in the United Kingdom. The proportion of China’s public-health expenditure in GDP and total fiscal expenditure is also much lower than that of the United States, the United Kingdom, and Japan. In fact, public-health expenditure accounts for the highest proportion of government expenditure in Japan and the United States. But in China, the share of public-health expenditure is less than that of infrastructure construction, education, national defense, urban and rural community affairs, and so on. In addition, compared with the three developing countries, the public-health expenditure level of China is slightly better than that of India, and not as good as Russia and Thailand. The data of 2018 in Table 3 also shows a similar result, that is, the level of public-health expenditure in China is very low and much lower than that in developed countries. This similar result also implies that the Chinese government has not significantly increased its investment in public health in recent years, nor has it paid more attention to public-health affairs.

The results of Wang [27] show that the deficiency of public-health expenditure has undergone a great improvement in China, and the public-health expenditure level in some provinces has even exceeded that in developed countries. But this result is opposite to Wang’s [27]. Even Beijing and Shanghai, which have the highest public-health expenditure levels in China, have spending levels far lower than those of developed countries (see Table 2 and Table 3). As Schwartz and Evans [38] points out, although the Chinese government has paid more attention to public-health expenditure after the SARS (severe acute respiratory syndrome) epidemic in 2003, it is still not enough.

(2) The Chinese government attaches less importance to public health affairs than other countries do. Firstly, the share of public-health expenditure in fiscal expenditure in China accounts for about for 1/4 of that in the United States and the United Kingdom, and 1/6 of that in Japan. It is slightly better than India, and not as good as Russia and Thailand (see Table 3). Secondly, the share of public-health expenditure in fiscal expenditure in most countries shows an upward trend, but that of China decreases by 0.26%, from 2014–2018. Thirdly, the proportion of China’s public-health expenditure in GDP is also significantly lower than that of the three developed countries, Thailand, and Russia, and has decreased slightly from 2014 to 2018. As is known to all, the tax burden, fiscal expenditure, and GDP of China have all increased sharply in recent years, but the proportion of public-health expenditure in fiscal expenditure and GDP has decreased. It implies that the Chinese government has not paid enough attention to public-health expenditure in recent years.
(3) In cases where there was insufficient attention to major public health emergencies both in quantity and quality, COVID-19 took advantage of such vulnerability and swept over the masses. As is shown in Table 1, it is apparent that Chinese government spending on prevention and control of diseases, major public-health service projects, and public-health emergency treatment is quite low—especially for the public-health emergency treatment, which accounts for less than 0.1% of the public-health expenditure on average in 2014–2018. And this expenditure item shows a fluctuating decline, from 0.8 billion yuan in 2014 to 0.68 billion yuan in 2018. These data reflect the inadequacy of the spending on major public-health emergencies in quantity.

Moreover, the prevention and control system of major public-health emergencies is an important part of public-health management. If this system is inefficient, the effectiveness of public-health management will accordingly decrease to a great extent. In fact, the inefficiency of this system in China, which reflects the inadequacy in quality, is crucially to blame for the widespread of COVID-19. The details follow.

Firstly, low efficiency is manifested in the information disclosure mechanism of major public-health emergencies. Some doctors had posted the information of novel coronavirus pneumonia on the Internet when the epidemic had not yet spread, including Dr. Li Wenliang, but they were punished by the local police for the reason of “spreading rumors” at the end of December 2019. It means that the local government had chosen to hide the disease, and in doing so planted the seeds of COVID-19 epidemic at that time.

Secondly, the response and decision-making mechanisms are inefficient. The earliest COVID-19 case could date back to early December 2019, while Wuhan suffered lockdown at January 23rd 2020. It indicates that the governments’ responses and decisions took more than one month, thus missing the best time for lockdown, and finally caused the breakout of COVID-19 with the beginning of Chinese Spring Festival transportation. In China, local government undertakes the main responsibilities of public health supervision, and epidemic prevention and control, but the permission of central government is needed to disclose the epidemic situation and enact city lockdown. This is the main reason why the response and decision-making mechanisms are inefficient.

Thirdly, the governmental primary medical institutions haven’t played their due role. Governmental medical institutions are classified into two categories in China, namely governmental primary medical institutions and governmental hospitals, to construct the system of tiered medical services. But this system fails to produce a marked effect. At the beginning of the epidemic in Wuhan, a large number of suspected patients rushed to the governmental hospital, and a considerable part of those had fevers caused by common colds. Due to the lack of tiered medical services and panic about the epidemic situation, these suspected COVID-19 patients flocked to large hospitals rather than primary medical institutions. This phenomenon is called “a run on medical resources”, which not only increases the risk of cross-infection and the burden of medical staff, but also wastes medical resources, making it difficult to offer timely support for patients who are really infected with COVID-19. This is an important reason for the rapid outbreak of epidemic in a short period in the hardest-hit city, Wuhan.

Fourthly, the Center for Disease Control and Prevention (CDCP) in China is not able to play an effective role in disease prevention and containment in reality. The CDCP is a public welfare institution with the main business of disease information collection and analysis, disease detection, and disease prevention and control research. As a public welfare institution, the CDCP finds it difficult to effectively play the role of “scout” and “sentry” when carrying out disease-control work with administrative behavior characteristics, especially when it faces a major public-health emergency like COVID-19. In addition, the CDCP has no right to order and coordinate medical institutions and other professional public health institutions at the same level.

Last, public-health emergency management mechanism is still weak. For example, the shortage of protective clothing and masks reflects the unsound reserve system of basic public-health emergency materials, and the deficient response and disposal capacity. Additionally, the number of patients in Hubei Province is very large, but the central government has not well-coordinated the public-health resources, which means that the stocks of medical masks, protective clothing, and other medical resources are still not enough to meet the actual needs of the patients, resulting in a higher mortality rate than in other provinces.

(4) Chinese public-health expenditure shows an increasing trend in recent years, but the increasing rate is too low, and lower than that of GDP and fiscal expenditure. With an average annual growth rate of 8.90%, Chinese public-health expenditure has increased from 864.75 billion yuan in 2014 to 1202.05 billion yuan in 2018 (see Table 1). But the proportion of public-health expenditure in GDP and fiscal expenditure shows a trend of first rising and then decreasing, and it has decreased in general. It means that despite rapid economic growth and government spending increasing, residents in China do not enjoy most of the dividends of economic growth in public health and medical services. As mentioned above, Chinese governments have invested a lot of dividends from economic growth into infrastructure construction in order to seek higher economic growth rate. According to the experience of the COVID-19 epidemic, it is also an important way to ensure stable economic growth by improving the public-health expenditure in quantity and quality, continuously perfecting the public-health system and policy, and preventing the recurrence of such major epidemics.
(5) Although the Chinese government inclines the public-health expenditure to the poor provinces in central and western regions, the imbalance and inequity in the allocation of public-health resources among regions are still expanding. China has carried out a comprehensive reform to alleviate the increasingly obvious inequities in public-health and medical services among regions, which has made public-health expenditure increase significantly in poor provinces from the central and western regions (see Table 2). The per capita public-health expenditure has increased in all 31 provinces of China since 2008. Among these provinces, Hainan, Jiangxi, Guizhou, and Guangxi are the top four provinces in terms of per capita public-health expenditure growth, with a growth rate of more than 400%, all of which are relatively poor provinces. In addition, the proportion of provincial public-health expenditure in provincial GDP and local fiscal expenditure has also increased significantly in central and western provinces. Obviously, the Chinese central government has invested a large amount of funds into the central and western provinces by transfer payment, which causes a large growth in public-health expenditure in these provinces.

**Figure 4:**
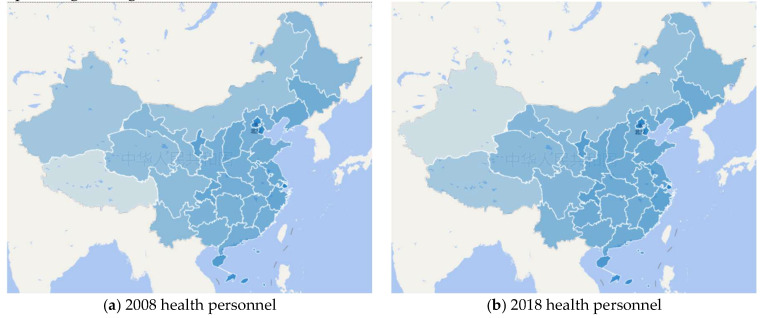

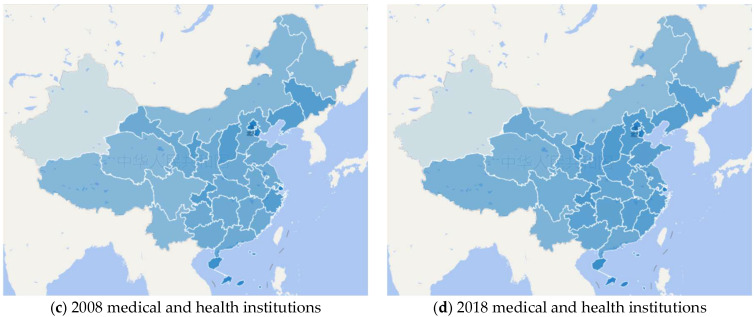
Geographical distribution of public-health resources. Note: (1) (**a**,**b**) respectively show the geographical distribution of health personnel in 2008 and 2018. And the health personnel is measured by the quantity of health personnel per thousand square kilometers per million people, namely “health personnel/ (thousand kilometers^2^• million people)”. (2) (**c**,**d**) respectively show the geographical distribution of medical and health institution in 2008 and 2018. The medical and health institution is measured by the quantity of medical and health institution per thousand square kilometers per million people, namely “medical and health institution/ (thousand kilometers^2^• million people)”. (3) The darker the blue, the more abundant the public-health resources in that area. (4) These figures above are made by the authors, and there is no copyright issue.

However, it is still difficult to alleviate the imbalance and inequality of public-health resources allocation only by increasing the public-health expenditure in central and western regions. As is shown in Figure 4, the resources of medical and health institutions are measured by the quantity of medical and health institutions per thousand square kilometers per million people, namely “medical and health institution/(thousand kilometers^2^• million people)”. Medical and health institutions include hospitals, primary medical and health institutions, disease control and prevention centers, and hygiene supervision institutions. Health personnel resources are measured by the quantity of health personnel per thousand square kilometers per million people, namely “health personnel/(thousand kilometers^2^• million people)”. The health personnel include occupational doctors, occupational assistant doctors, nurses, pharmacists, rural doctors, and hygiene management personnel. Public-health resources as a whole shows a decreasing trend from east to west both in 2008 and 2018. In 2008, Beijing, Shanghai, and Tianjin were the top three provinces with the most abundant public-health resources, while Tibet, Xinjiang, and Inner Mongolia were the bottom three. The ranking of public-health resources remained similar in 2018. Moreover, imbalance and inequity in the allocation of public-health resources among regions are still expanding. For example, the quantity of health personnel per thousand square kilometers per million people in Beijing has increased from 517.83 in 2008 to 724.40 in 2018, while that in Tibet has increased from 2.62 to 4.51 in the same period. It means that the gap of public-health resources between Beijing (with the most abundant public-health resources and Tibet (with the least public-health resources) continued expanding during 2008–2018.

(6) There are a lot of wasted resources in the public-health system, which seriously reduces the efficiency of public-health expenditure in China. Although Table 3 shows that the public-health expenditure level of China is higher than that of India, it does not mean that expenditure efficiency is higher, or that the residents’ sense of gain is higher in China, because there is a huge waste of resources in China’s medical service and insurance system. The Chinese government has carried out the market-oriented reform of governmental hospitals, and requires these hospitals to be responsible for their own profits and losses. Thus, the responsibility for making profits is mainly shared by all doctors, which will lead to doctors treating minor diseases in the way of treating serious diseases, requiring patients to participate in various physical tests, prescribing a large number of safe, inefficient, and high-profit drugs, and finally making the patients and the medical insurance system pay the bill. At the same time, many governmental hospitals increase their income through economies of scale; for example, the number of beds in the First Affiliated Hospital of Zhengzhou University is more than 10,000. Such a large-scale hospital is almost unimaginable in the United Kingdom and the United States, but there are many in China. Although it brings convenience for residents to get medical services, it not only causes a great waste of resources, but also does not bring residents a higher sense of gain, because such a large hospital needs residents and the medical insurance system to bear most of the costs. The ultimate result is a great waste of resources, medical insurance fund deficit, and a low sense of patient acquisition, which all reflect the low efficiency of Chinese public-health expenditure.

## 5. Conclusions

After clarifying the definition and items of public-health expenditure, this paper objectively evaluates the performance of the Chinese government in public health by measuring Chinese public-health expenditure at the national and provincial levels followed by international comparison. In general, the Chinese government hasn’t done a good job in public health. Specifically, (1) the level of public-health expenditure in China is relatively low, and much lower than that of developed countries; (2) Chinese governments have not paid enough attention to the prevention and control of major public-health emergencies, which may be an important reason for the outbreak of COVID-19; (3) Chinese public-health expenditure shows a fluctuating growth trend, but the growth rate is relatively low, and is lower than that of GDP and total fiscal expenditure; (4) although the Chinese government inclines the public-health expenditure to the poor provinces in central and western regions, the imbalance and inequity of public-health resources allocation are still expanding among regions; (5) there are a lot of wasted resources in the public-health system, which seriously reduces the efficiency of public-health expenditure in China.

Based on the conclusions above, this paper suggests that the Chinese government should improve the “quantity” and “quality” of public-health expenditure in the following aspects:

Firstly, it’s better to increase the scale of public-health expenditure and ensure a reasonable expenditure structure. On the one hand, the Chinese government should increase public-health expenditure and its proportion in government expenditure and GDP, so that its growth rate can keep pace with, or even exceed, the economic growth rate. On the other hand, the Chinese government spends most of its public-health expenditure on medical services, such as medical insurance subsidies and governmental medical institutions, while neglecting narrow-caliber public-health expenditure, such as health supervision, public-health emergency treatment, and so on (see Table 1). Therefore, the Chinese government should ensure the sustainable growth of public-health expenditure and substantially increase the NPHE, so as to achieve the goal of increasing the scale and rationalizing the structure at the same time.

Secondly, the Chinese government should pay more attention to the prevention and control of major public health emergencies, and gradually form a set of comprehensive and scientific systems. First of all, the scale and growth of the spending on major public-health emergencies must be guaranteed. Second, the CDCP should undertake the responsibility of epidemic research and early warning. Specifically, the CDCP needs to study the current epidemic virus and the possible pathogenic source in industry and chemistry, and then come up with the corresponding prevention and solution measures. Third, it is necessary to establish an inter-governmental cooperative decision-making and response mechanism. The central government and local government should respectively undertake cross-regional and inner-regional major public health emergencies. For example, when the epidemic spread in Wuhan in the first place, the local government should have had the right to make the decision of the news disclosure and city lockdown. Fourth, it is better to establish the strategic reserve mechanism of national medical materials, so as to mobilize medical materials quickly and in a timely manner.

Thirdly, we advise that the Chinese government should promote the balance and equity of public-health resource allocation among provinces. On one hand, central government should continuously increase the transfer payment to the provinces with poor public-health resource. On the other hand, the governments of poor provinces in central and western regions should strengthen local economic development and financial resources construction to provide sufficient funds for public-health affairs.

Lastly, there are some suggestions on reducing the waste of medical resources. As mentioned above, the Chinese government has not spent enough on governmental hospitals, leading to the cases where hospitals have to make profits by making patients do a lot of unnecessary physical examinations and prescribing more drugs, and finally causing a serious waste of medical resources. Therefore, it is necessary to substantially increase the expenditure of governmental hospitals. In addition, the Chinese government should further promote the reform of the medical and health system, and gradually perfect the medical security system, so that governmental hospitals can become a “governmental welfare institution”, rather than a “profit-making institution”.

There are two points need to be further studied. Firstly, why is the imbalance of public-health resources distribution among regions in China still not effectively alleviated? This is the case even though the Chinese government has significantly increased public-health expenditure in the central and western provinces in the past decade or so. Secondly, is excessive fiscal decentralization an important reason for low public-health expenditure level in China? As the existing literatures show, fiscal decentralization will intensify the competition among local governments and distort the structure of fiscal expenditure. Specifically, local governments prefer to invest in projects with direct economic benefits, such as infrastructure construction, rather than use fiscal funds for projects that lack direct economic benefits, such as public health.

## Figures and Tables

**Table 1:** Chinese public-health expenditure at national level from 2014–2018.

Public-Health Expenditure		2014	2015	2016	2017	2018
Items (unit: billion CNY)	Governmental hospitals	137.11	172.67	207.51	219.35	229.47
	Governmental primary medical institutions	93.80	110.24	121.06	132.52	137.91
	Prevention and control of infectious diseases	23.64	28.01	31.29	34.20	37.34
	Hygiene supervision	6.68	7.71	8.39	9.31	10.08
	Maternal and child health care	10.95	13.71	16.65	19.97	19.40
	Mental health care	0.54	0.76	0.79	0.85	0.99
	Emergency medical treatment	1.91	2.26	2.36	2.71	3.06
	Public blood banks	4.78	5.59	6.26	7.36	7.63
	Major public-health service projects	27.59	27.66	27.76	28.73	28.80
	Public health emergency treatment	0.80	0.61	0.62	0.85	0.68
	Traditional Chinese medicine	2.44	3.23	3.86	4.19	4.95
	Food and drug supervision	24.28	33.67	39.02	43.63	45.69
	Medical insurance subsidy	462.23	541.65	595.33	502.41	548.27
	Medical assistance	21.29	24.06	26.69	32.09	46.97
	Other basic public-health services	46.73	59.07	65.65	71.05	80.81
Total public-health expenditure (unit: billion CNY)		864.75	1030.87	1153.24	1109.20	1202.05
Per capita public-health expenditure (unit: CNY)		632.21	749.93	834.04	797.94	861.45
Total public-health expenditure/GDP (%)		1.35	1.50	1.56	1.35	1.34
Total public-health expenditure/fiscal expenditure (%)		5.70	5.86	6.14	5.46	5.44

Note: CNY is short for the Chinese Yuan.

**Table 2:** Chinese public-health expenditure at provincial level.

Province	Per Capita Public-Health Expenditure (Unit: CNY)			Public-Health Expenditure/GDP			Public-Health Expenditure/Total Fiscal Expenditure		
	2008	2014	2018	2008	2014	2018	2008	2014	2018
Beijing	819.03	1283.63	1750.54	1.30%	1.28%	1.14%	7.40%	6.05%	5.05%
Tianjin	356.46	917.27	950.68	0.62%	0.87%	1.11%	4.83%	4.75%	4.78%
Hebei	172.04	515.91	703.94	0.75%	1.29%	1.64%	6.39%	8.12%	6.88%
Shanxi	209.62	569.59	742.87	0.98%	1.62%	1.73%	5.44%	6.72%	6.45%
Inner Mongolia	244.76	773.76	958.29	0.70%	1.09%	1.50%	4.11%	4.99%	5.03%
Liaoning	194.44	529.56	618.86	0.61%	0.81%	1.15%	3.90%	4.58%	5.05%
Jilin	217.70	637.55	800.17	0.93%	1.27%	1.92%	5.04%	6.02%	5.71%
Heilongjiang	187.45	521.50	613.79	0.86%	1.33%	1.80%	4.65%	5.82%	4.95%
Shanghai	571.13	929.44	1492.17	0.87%	0.95%	1.00%	4.71%	4.57%	4.33%
Jiangsu	191.46	599.59	807.82	0.48%	0.73%	0.70%	4.58%	5.63%	5.58%
Zhejiang	274.12	669.85	839.79	0.67%	0.92%	0.83%	6.47%	7.14%	5.58%
Anhui	169.26	596.28	762.93	1.17%	1.73%	1.42%	6.30%	7.74%	7.34%
Fujian	204.09	655.00	862.31	0.69%	1.03%	0.88%	6.53%	7.51%	7.03%
Jiangxi	174.82	634.56	969.12	1.10%	1.83%	1.98%	6.36%	7.41%	7.95%
Shandong	149.11	527.25	677.83	0.45%	0.87%	1.02%	5.19%	7.17%	6.74%
Henan	154.28	543.65	744.11	0.81%	1.47%	1.43%	6.38%	8.50%	7.75%
Hubei	166.49	587.18	748.63	0.84%	1.25%	1.05%	5.76%	6.91%	6.10%
Hunan	137.30	534.59	699.35	0.76%	1.33%	1.33%	4.96%	7.15%	6.45%
Guangdong	203.33	618.43	954.44	0.55%	0.97%	1.08%	5.32%	7.22%	6.88%
Guangxi	163.56	637.48	853.60	1.12%	1.93%	2.14%	6.07%	8.68%	7.92%
Hainan	194.85	836.12	1189.99	1.11%	2.15%	2.26%	4.65%	6.83%	6.57%
Chongqing	181.90	702.33	924.62	0.89%	1.47%	1.33%	5.08%	6.33%	6.32%
Sichuan	176.41	611.01	812.54	1.14%	1.74%	1.58%	4.87%	7.30%	6.98%
Guizhou	187.54	735.19	1029.69	1.89%	2.78%	2.41%	6.40%	7.27%	7.37%
Yunnan	230.22	637.10	916.60	1.84%	2.34%	2.12%	7.11%	6.75%	7.29%
Tibet	559.93	1318.02	2391.56	4.14%	4.51%	5.31%	4.30%	3.50%	4.17%
Shaanxi	210.84	706.59	906.59	1.07%	1.51%	1.46%	5.49%	6.72%	6.61%
Gansu	228.62	670.82	914.77	1.84%	2.54%	2.98%	6.02%	6.83%	6.39%
Qinghai	445.13	1172.96	1806.70	2.42%	2.96%	3.96%	6.78%	5.05%	6.61%
Ningxia	276.86	842.86	1180.35	1.42%	2.02%	2.31%	5.27%	5.54%	5.72%
Xinjiang	275.18	753.68	885.20	1.40%	1.85%	1.72%	5.54%	5.18%	4.39%

Note: “Province” here indicates municipalities directly under the central government (i.e., Shanghai Municipality, Beijing Municipality) and autonomous regions (i.e., Tibet Autonomous Region) in China.

**Table 3:** The public-health expenditure of seven countries.

	Per capita Public-Health Expenditure (Local Currency/USD)		Public-Health Expenditure /Total Fiscal Expenditure		Public-Health Expenditure /GDP	
	2014	2018	2014	2018	2014	2018
The US	4069	4943	20.94%	22.36%	7.44%	7.89%
The UK	2075/3415	2302/3071	19.49%	20.22%	7.27%	7.23%
Japan	301,314/2844	325,277/2946	36.25%	37.23%	7.29%	7.37%
China	632/103	861/130	5.70%	5.44%	1.35%	1.34%
Russia	17,278/450	22,815/364	7.83%	9.25%	1.35%	3.05%
India	1173/19	—	4.29%	—	1.18%	—
Thailand	4061/125	4871/151	11.15%	11.31%	2.86%	3.03%

Note: (1) Considering the impact of exchange rate changes, per capita public-health expenditure is measured in local currency and US dollar (local currency/US dollar). (2) India’s public-health expenditure data in 2018 cannot be found in the database of India’s Ministry of Finance.
